# Case Report: Air–fluid point: a specific ultrasound sign for the diagnosis of hydropneumothorax in newborn infants

**DOI:** 10.3389/fped.2025.1637262

**Published:** 2025-09-16

**Authors:** Jing Liu, Peng Jiang

**Affiliations:** Department of Neonatology and NICU, Beijing Obstetrics and Gynecology Hospital, Capital Medical University, Beijing Maternal and Child Health Care Hospital, Beijing, China

**Keywords:** air–fluid point, hydropneumothorax, lung ultrasound, newborn infant, pneumothorax, pleural effusion

## Abstract

Currently, lung ultrasound (LUS) is widely used worldwide for the diagnosing conditions such as neonatal pneumothorax (PTX) and pleural effusion, which are both common conditions during the neonatal period. However, the coexistence or simultaneous occurrence of both conditions, namely, hydropneumothorax, is relatively rare in clinical practice. Ultrasound enables a reliable diagnosis and can be used to guide and monitor appropriate treatment. In this case report, we describe a new ultrasound sign, the air–fluid point, which we propose as a specific sign for the diagnosis of neonatal hydropneumothorax.

## Introduction

In recent years, lung ultrasound (LUS) has been successfully applied in the diagnosis and differential diagnosis of various lung diseases in newborn infants, and the technology has become well-established (1–5). LUS plays an irreplaceable and important role in the treatment of severe and complex lung diseases (6). Currently, LUS is widely used worldwide, and in some institutions, it has even replaced chest x-ray (CXR) as the routine imaging modality used in neonatal intensive care units (7). Pleural effusion and pneumothorax (PTX) are common conditions in newborns, and ultrasound can easily yield a clear diagnosis and be used to guide and monitor appropriate treatment (8–10). However, the simultaneous occurrence of both conditions, namely, hydropneumothorax, is a relatively rare condition in clinical practice. The ultrasound manifestations of neonatal hydropneumothorax have not yet been reported in the literature. The patient whose case is presented here was admitted to the neonatal intensive care unit (NICU) because of premature birth, congenital pleural effusion, and respiratory distress. Pneumothorax subsequently occurred, leading to a diagnosis of hydropneumothorax through ultrasound examination. In this report, the ultrasound imaging characteristics of hydropneumothorax are introduced to expand the scope of LUS applications.

## Case presentation

**Figure 1:**
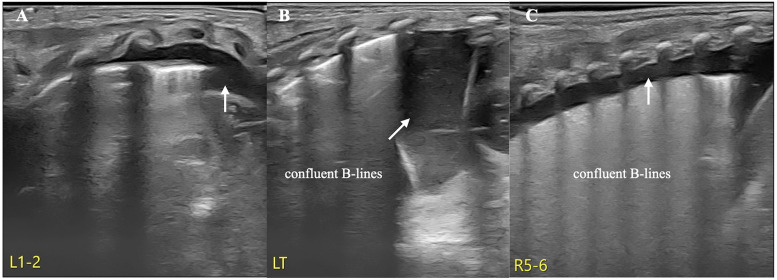
TTN and pleural effusion. LUS revealed anechoic areas in both thoracic cavities and significantly confluent B-lines in both lung fields but no lung consolidation with air bronchograms. Therefore, the patient was diagnosed with congenital pleural effusion and TTN (**A**: left anterior chest, **B**: left posterior chest, **C**: right posterior chest).

A female infant with a gestational age of 31 weeks and 2 days was delivered via emergency cesarean section owing to unknown causes of fetal heart rate reduction. She was admitted to the NICU because of premature birth and mild respiratory difficulties shortly after birth. LUS examination on admission revealed anechoic areas in the bilateral thoracic cavities and a significant number of confluent B-lines in both lungs but no lung consolidation with air bronchograms. Therefore, the patient was initially diagnosed with congenital pleural effusion and transient tachypnea of the newborn (TTN) according to the LUS findings, and noninvasive respiratory support was administered to the infant (Figure 1) (informed consent to publish the images and videos was obtained from the parents).

To determine the characteristics of the pleural effusion, thoracentesis was performed, resulting in drainage of pale yellow fluid from both sides of the thoracic cavity. Routine examination of the pleural fluid revealed a total cell count of 3,567 × 10^6^/L, of which neutrophils accounted for 98.8% of the total cells. The results of the Leve-Ivanoff test were negative. The specific gravity of the pleural fluid was 1.020. Biochemical analysis revealed the following: 3.94 mmol/L of Glu, 112 IU/L of LDH and 23.6 g/L of protein. The results of the chylous test were positive. Both the pleural fluid and blood cultures were negative. The infant was confirmed to have congenital chylous pleural effusion.

**Figure 2:**
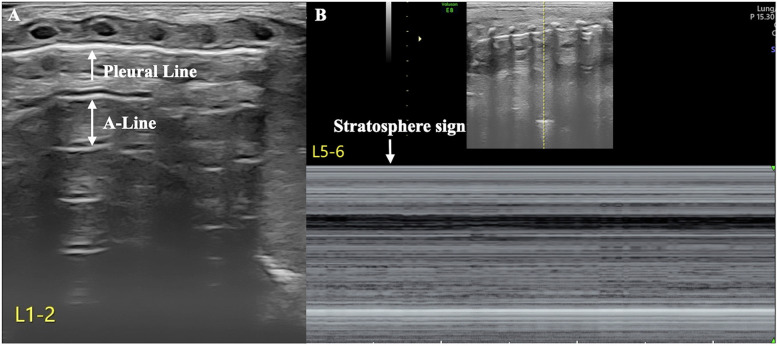
Severe pneumothorax in the left thoracic cavity. LUS revealed clear pleural lines and A-lines in the anterior chest, axilla, and posterior chest of the left lung on B-mode ultrasound. M-mode ultrasound revealed the stratosphere sign, and real-time ultrasound revealed disappearance of lung sliding (Supplementary Video S1), suggesting a large amount of pneumothorax in the left thoracic cavity (**A**: B-mode ultrasound, **B**: M-mode ultrasound).

**Figure 3:**
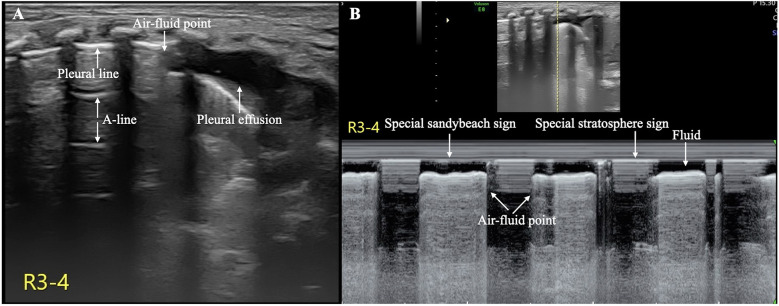
Hydropneumothorax in the right thoracic cavity. The pleural line and A-line are clearly displayed in the upper lung field, and an anechoic dark area is observed in the lower lung field on B-mode ultrasound **(A)** On real-time ultrasound, a demarcation point between the gas and fluid interfaces is observed; this kind of demarcation point is referred to as the air–fluid point in this paper (Supplementary Video S2). M-mode ultrasound revealed the air–fluid points as alternating points of the characteristic sand on the beach sign and the characteristic stratosphere sign **(B)** These findings confirmed that both pleural effusion and pneumothorax coexisted (i.e., hydropneumothorax).

The patient's condition was relatively stable on the first and second days after birth, with noninvasive respiratory support maintaining stable vital signs. However, her respiratory distress suddenly worsened on the third day after birth, and the oxygen concentration required invasive respiratory support to maintain normal blood oxygen saturation without any obvious triggers. LUS reexamination revealed clear pleural lines and A-lines on B-mode ultrasound of the left anterior chest, axilla, and back. The stratosphere sign appeared on M-mode ultrasound. Real-time ultrasound revealed the disappearance of lung sliding. According to the diagnostic and grading criteria of PTX, this child meets the diagnostic criteria for severe PTX in the left thoracic cavity (9–11) (Figure 2, Supplementary Video S1). Moreover, ultrasound signs indicating a moderate degree of PTX were also observed in the anterior chest and the upper axillary region of her right thoracic cavity (9–11). In the lower field of the right axilla, an anechoic dark area was observed, and alternating visibility of the lung sliding was observed between the upper and lower fields on real-time ultrasound. Under M-mode ultrasound, these areas presented as alternating characteristics between the beach sign and the stratosphere sign, similar to the lung point (11, 12), confirming a small amount of pleural effusion combined with moderate pneumothorax, specifically hydropneumothorax (Figure 3, Supplementary Video S2).

Owing to the presence of pneumothorax on both sides of the thoracic cavity and the severity of the patient's condition, closed thoracic drainage was performed. Two days later, both the pneumothorax and pleural effusion had fully resolved. After continuing treatment for another two weeks, the infant was able to breastfeed normally, and her vital signs remained stable. There was no recurrence of pleural effusion or pneumothorax, and the patient was discharged from the hospital and fully recovered.

## Discussion

Chylous pleural effusion is the most common type of congenital pleural effusion observed in newborns, and injury to or disruption of the thoracic duct, which is responsible for chyle transport, is considered to be the major cause of chylothorax (13, 14). Although congenital chylothorax is not common, it can lead to a poor prognosis, with a mortality rate as high as 28% (14). Because of the rapid growth of chylous effusion and the frequent need for continuous closed thoracic drainage via thoracentesis in severe cases, we cannot rule out that bilateral pneumothorax may have been caused by accidental injury to the pleura during the thoracic puncture procedure. However, inadequate respiratory support and the resulting undertreatment of respiratory distress are more likely to be the main causes. Owing to timely monitoring, detection, and treatment with point-of-care ultrasound, a satisfactory outcome was achieved for the infant.

PTX is a common, critical condition encountered in the NICU, with an incidence rate of 1%–2% in term infants and 6% in premature infants with respiratory distress, according to the literature (15, 16). The development and application of LUS have revealed that the incidence of PTX in NICU patients with high-risk factors may be as high as 10% (16). The occurrence of PTX is associated with a 5.27-fold increase in neonatal mortality and a 30% higher incidence of bronchopulmonary dysplasia (BPD) among surviving premature infants. Therefore, timely, rapid and accurate diagnosis, as well as prompt and correct puncture treatment, are essential in patients with risk factors (5, 17).

Ultrasound is a simple and effective imaging procedure for the diagnosis of pleural effusion and pneumothorax (8–11). However, when both conditions occur simultaneously, ultrasound techniques for diagnosing these conditions and identifying specific ultrasound imaging features observed in the field of neonatology have not yet been explored. The infant in our case study showed signs of PTX, such as clear pleural lines and A-lines, as well as pleural effusion indicated by the absence of echoic dark areas, simultaneously on B-mode ultrasound. On real-time ultrasound, gas and fluid alternate at their interface, and this type of air–fluid demarcation point is referred to as the “air–fluid point” in this paper. Under M-mode ultrasound, gas signs (a characteristic stratospheric sign) and liquid signs (a characteristic sand on the beach sign) alternately emerge, and the point where they alternate is called the “air‒fluid point”. Therefore, although hydropneumothorax is extremely rare in clinical practice, accurate diagnosis via ultrasound is feasible provided that technicians are highly skilled in ultrasound diagnostic techniques for pneumothorax and effusion. Notably, the “normal” findings on B-mode ultrasound for an infant with breathing difficulties do not necessarily indicate true normality; it is necessary to observe pneumothorax under real-time ultrasound and, if necessary, to conduct M-mode ultrasound for verification.

In conclusion, this article presents a typical case in which the “air-fluid point” was introduced as a specific ultrasound sign to aid in the diagnosis of neonatal hydropneumothorax. Of course, its reliability still needs further verification through the accumulation of more cases, particularly given potential differences in ultrasound manifestations at different gas-to-liquid ratios.

## Data Availability

The original contributions presented in the study are included in the article/Supplementary Material, further inquiries can be directed to the corresponding author.

## Supplementary material

Supplementary Video S1 PTX manifestations on real-time ultrasound. On real-time ultrasound, the pleural lines and A-lines are clearly visible, but lung sliding disappears. These signs are the most important indicators for the diagnosis of PTX via ultrasound.

Supplementary Video S2 The air–fluid point: A specific ultrasound sign of hydropneumothorax. On real-time ultrasound, the demarcation point where gas and fluid alternate at their interface can be observed; this point is referred to as the air‒fluid point in this paper.
